# Metastasis of Neuroendocrine Tumors Are Characterized by Increased Cell Proliferation and Reduced Expression of the ATM Gene

**DOI:** 10.1371/journal.pone.0034456

**Published:** 2012-04-02

**Authors:** Jeeyun Lee, Chang Ohk Sung, Eui J. Lee, In-Gu Do, Hee-Cheol Kim, Seong Hyeon Yoon, Woo Yong Lee, Ho Kyung Chun, Kyoung-Mee Kim, Young Suk Park

**Affiliations:** 1 Division of Hematology-Oncology, Departments of Medicine, Sungkyunkwan University School of Medicine, Seoul, Korea; 2 Pathology, Sungkyunkwan University School of Medicine, Seoul, Korea; 3 Research Institute, Incheon St. Mary Hospital, Catholic University of Korea, Seoul, Korea; 4 Samsung Cancer Research Institute, Sungkyunkwan University School of Medicine, Seoul, Korea; 5 Surgery, Samsung Medical Center, Sungkyunkwan University School of Medicine, Seoul, Korea; Florida International University, United States of America

## Abstract

**Purpose:**

Gastroenteropancreatic neuroendocrine tumors (GEP-NETs) are rare group of tumors with a wide spectrum of clinical behavior. However, there are no known clinically relevant biomarkers to predict metastasis.

**Experimental Design:**

To investigate differential gene expression signatures of metastatic *vs* non-metastatic NETs, we studied cell cycle regulatory genes in 19 metastatic and 22 non-metastatic colorectal NETs by PCR arrays. Immunohistochemistry (IHC) and quantitative real-time RT-PCR were performed to verify the results and another set of 38 GEP-NETs were further studied for validation.

**Results:**

We first delineated six candidate genes for metastasis including *ATM*, *CCND2*, *RBL2*, *CDKN3*, *CCNB1*, and *GTSE1*. ATM was negatively correlated with metastatic NETs (*p*<0.001) with more than 2-fold change compared to non-metastatic NETs. Overexpression of ATM protein by IHC was strongly correlated with high ATM mRNA levels and low Ki-67 labeling index. Patients with ATM-negativity by IHC showed significantly decreased overall survival than patients with ATM-positivity (median OS, metastatic *vs* non-metastatic NETs; 2.7 years *vs* not reached; *p* = 0.003) and 85.7% of metastatic NETs were ATM-negative. In another validation set of GEP-NETs, decreased mRNA of *ATM* gene was associated with metastasis and remained significant (*p* = 0.023).

**Conclusions:**

ATM down-regulation was strongly associated with metastatic NETs when compared with non-metastatic NETs and ATM may be a potential predictive marker for metastasis as well as a novel target in metastatic GEP-NETs.

## Introduction

Gastroenteropancreatic neuroendocrine tumors (GEP-NETs) are a heterogeneous group of malignancies derived from neuroendocrine cell compartments in various organs including pancreas, colorectum, biliary tract, and stomach. Data from recent population-based studies demonstrate a significant increase in the reported incidence of NETs over time and the incidence ranges from 2.5 to 5 cases per 100,000 in Caucasian population [1]–[4]. The increase in incidence is likely attributable, in part, to increasing awareness, improved diagnostic strategies for NETs, and possibly other undetermined environmental and genetic factors [5].

NETs are a broad family of tumors with a wide range of clinical presentations and outcomes. Given the biological diversity of GEP-NETs, the treatment of GEP-NETs is now becoming more optimized according to subgroups. Recently, WHO classified NETs as well-differentiated neuroendocrine tumor, well-differentiated neuroendocrine carcinoma, and poorly differentiated neuroendocrine carcinoma [6]–[8]. Nevertheless, there are no candidate biological markers to guide a novel targeted therapeutic approach, partially owing to the fact that little is known about the underlying mechanisms that regulate development and progression of NETs. Several clinicopathological factors have been reported to predict poor clinical outcome in NETs. These include histologic grade, disease stage, sex, race, age, year of diagnosis, tumor size, and proliferation indices including Ki-67 labeling index and mitotic count [1], [9]–[11]. In addition to tumor size, proliferation index are one of the most important factors associated with metastasis. These findings suggest that certain defects in cell cycle regulatory mechanism are associated with cell proliferation and may predict malignant behavior of NETs. In this study, we studied gene expression signatures of cell cycle regulatory genes in metastatic and non-metastatic colorectal NETs to delineate candidate genes which may predict metastatic behavior.

## Materials and Methods

### Patient samples

Cases of colonic (n = 4) and rectal (n = 15) metastatic NETs (n = 19) were selected from July 1, 1996 to December 31, 2008 who were treated at Samsung Medical Center. This retrospective study was approved by the institutional review board at Samsung Medical Center and was waived for written informed consent form. Case selection criteria for metastatic and non-metastatic NETs were as following: complete demographic data, treatment record (Appendix S1), availability of fresh frozen tissues and paraffin-embedded tissue blocks. For control cases, colorectal NETs (n = 22) which were localized initially at diagnosis and during the median follow-up period of 6.8 years (range, 3.9–10.4 years) were selected from the database. To verify results and further investigate, another set of 38 metastatic (n = 20) and non-metastatic (n = 18) GEP-NETs from pancreas (n = 32), stomach (n = 3), ampulla of Vater (n = 1) and rectum (n = 2) were also analyzed.

A histopathologic review was conducted by two pathologists (C.O.S and K.M.K) to verify the diagnosis and histopathology of all cases. All study materials were obtained under the protocol approved by the Institutional Review Board of Samsung Medical Center. Histological grade was categorized as following; carcinoid tumor or well differentiated NET was classified as G1 tumor, atypical carcinoid or well differentiated neuroendocrine carcinoma was classified as G2 tumor, and poorly differentiated neuroendocrine carcinoma was classified as G3 tumor [12]. Among 41 NETs studied, 36 were G1 and 5 were G2 tumors. G3 tumors were excluded from case selection. The demographic characteristics of cases and controls are outlined in Table 1.

**Table 1:** Characteristics of the cases used in the analysis.

Characteristic	Metastatic NET (n = 19)	non-metastatic NET (n = 22)
Age, years		
Median	62	53
Range	34–79	30–72
Sex		
Male	11 (41%)	16 (59%)
Female	8 (57%)	6 (43%)
Location		
Rectum	15 (41%)	22 (59%)
Colon	4 (100%)	0 (0%)
Histology grade		
G1	14 (39%)	22 (61%)
G2	5 (100%)	0 (0%)
Treatment		
Biopsy	11 (39%)	7 (61%)
Local excision	3 (17%)	15 (83%)
Radical resection	5 (100%)	0 (0%)

NET, neuroendocrine tumor.

### Expression of cell cycle regulatory genes by PCR Arrays

All quantitative RT-PCR experiments were conducted with a Light Cycler 480 real-time PCR system (Roche Applied Science, Indianapolis, IN). For PCR array experiments, a RT^2^ Profiler Custom PCR Array was used to simultaneously examine the mRNA levels of the genes of 84 genes key to cell cycle regulation and five reference genes including 18SrRNA, HPRT, RPL13A, GAPDH, and β-actin, according to the protocol of the manufacturer (SuperArray Bioscience, Frederick, MD). Total RNAs from formalin-fixed paraffin-embedded (FFPE) tumor tissue was extracted using RT^2^ FFPE RNA extraction kit according to the manufacturer's instructions (SuperArray). Between 0.3 and 2.8 µg of total RNA was added to each well of the ReactionReady First Strand cDNA Synthesis Kit (SuperArray no. C-01; SuperArray). 5 ul of the first-strand cDNA synthesis reaction were pre-amplified by the RT^2^ PreAMP PCR Master Mix and RT^2^ FFPE PreAMP Primer Mix at 95°C for 10 min; 8 cycles of 95°C for 15 s, and 60°C for 2 min. The equivalent of 0.8 µl cDNA was added to each well of the PCR array. A total volume of 25 µl of PCR mixture, which included 12.5 µl of RT2 Real-Time SYBR Green/Fluorescein PCR master mix from SuperArray Bioscience, 11.5 µl of milliQ, and 1 µl of template cDNA, was loaded in each well of the PCR array. Arrays were run with the following parameters: cycle 1, 10 min at 95°C; and cycle 2, 15 sec at 95°C, followed by 1 min at 60°C (40 repeats), with optical data collection at 60°C for each repeat. This experiment was repeated three times. Data were analyzed using the comparative cycle threshold method with normalization of the raw data to 5 reference genes. Functional gene grouping of 84 genes is shown in Table 2.

**Table 2:** Functional grouping of 84 cell cycle regulatory genes used in PCR array analysis.

Functional group	Gene
G1 phase and G1/S transition	ANAPC2, CCND1, CCNE1, CDC34, CDK4, CDK6, CDKN1B, **CDKN3**, CUL1, CUL2, CUL3, SKP2
S phase and DNA replication	ABL1, MCM2, MCM3, MCM4, MCM5, PCNA, RPA3, SUMO1, UBE1
G2 phase and G2/M transition	ANAPC2, ANAPC4, DIRAS3, BCCIP, BIRC5, **CCNB1**, CCNG1, CCNH, CCNT1, CCNT2, CDK5R1, CDK5RAP1, CDK7, **CDKN3**, CKS1B, CKS2, DDX11, DNM2, GTF2H1, **GTSE1**, HERC5, KPNA2, MNAT1, SERTAD1
M phase	CCNB2, CCNF, CDC2, CDC16, CDC20, MRE11A, RAD51
Cell cycle checkpoint and cell cycle arrest	**ATM**, ATR, BRCA1, BRCA2, CCNG2, CDC2, CDC34, CDK2, CDKN1A, CDKN1B, CDKN2A, CDKN2B, **CDKN3**, CHEK1, CHEK2, CUL1, CUL2, CUL3, GADD45A, HUS1, KNTC1, MAD2L1, MAD2L2, NBN, RAD1, RAD17, RAD9A, RB1, RBBP8, TP53
Regulation of cell cycle	ABL1, ANAPC2, ANAPC4, DIRAS3, **ATM**, ATR, BCCIP, BCL2, BRCA2, **CCNB1**, CCNB2, CCNC, CCND1, **CCND2**, CCNE1, CCNF, CCNH, CCNT1, CCNT2, CDC16, CDC2, CDC20, CDK2, CDK4, CDK5R1, CDK6, CDK7, CDK8, CDKN1A, CDKN1B, CKS1B, DDX11, E2F4, GADD45A, KNTC1, MKI67, PCNA, RAD9A, RB1, SKP2, TFDP1, TFDP2
Negative regulation of cell cycle	**ATM**, BAX, BRCA1, CDKN2B, RBL1, **RBL2**, TP53

Bold indicate significant genes in this study.

### Cell cycle regulatory gene expression signature analysis using bioinformatic methods

Volcano plot was used to identify significant differential expression genes between metastatic NETs and non-metastatic NETs. Briefly, the differences (log_2_fold change) between the two groups were plotted on X-axis. The –log10 (*P* value) were plotted on the y-axis. A cutoff value of *P* value<0.01 and fold change >1.5 were used to determine the differential gene expression signature. Principal component analysis was performed using R program (version 2.12.0: http://www.r-project.org) with prcomp function.

### Immunohistochemistry

Immunohistochemistry (IHC) was performed for Ki-67 (clone MIB-1, DAKO M7240, dilution 1∶70) and Ataxia Telangiectasia Mutated (ATM) (mouse monoclonal, ab78, Abcam, Cambridge, MA, Cat No. ab78, dilution 2 ul/ml) on 3 µm sections from FFPE tissues and mounted on positively charged slides. Tissue sections on glass slides were deparaffinized in xylene, hydrated in descending concentrations of alcohol, and then washed in distilled water. Antigen retrieval was performed with a microwave for 5 minutes 2 times with 10 mM citrate buffer (pH 6.0) for Ki-67, and with 97°C for 20 minutes with Tris/EDTA buffer (pH 8.0) for ATM. The slides were washed in phosphate buffer for 3 times. The slides were incubated with primary antibodies for 60 minutes, and incubated with biotinylated antibodies (Envision plus, Dako, Carpinteria, CA, USA) for 25 minutes and 60 minutes, respectively. Endogenous peroxidase activity was blocked with 3% hydrogen peroxide in distilled water for 10 minutes. After washing, the slides were incubated with peroxidase-labeled streptavidin complex for 25 minutes. The slides were incubated in a solution of 3% diaminobenzidine for 20 minutes and counterstained with hematoxylin. To generate Ki-67 labeling proliferation index, image analysis was performed with the Applied Imaging Ariol SL-50 (Genetix, San Jose, USA). The immunostained slides were scanned under KiSight protocol for Ki-67 analysis. A first-scan pass at 1.25× objective magnification was performed to acquire a low-resolution image to recognize the tissue. Selection of regions of interest was performed by a pathologist. A second scan-pass was performed at 20× objective magnification to obtain a high resolution image. After training color and shape classifiers, the regions were selected for analysis. For Ki-67, more than 1,000 tumor cells were analyzed. On the basis of the intensity of staining and the nuclear morphology, the machine calculated the results as a percentage of positive cells. The ATM positivity was defined as greater than 5% ATM (+) tumor cells in whole tumor section area.

### RNA Extraction and Real-Time Quantitative PCR of ATM

RNAs from FFPE tumor tissue was extracted using the RNeasy FFPE Kit (QIAGEN GmbH, Hilden, Germany) according to the manufacturer's instructions. ATM mRNA quantification was measured by real-time PCR based on TaqMan Gene Expression Assays (Applied Biosystems) as previously described [13]. Glyceraldehyde-3-phosphate-dehydrogenase (GAPDH) mRNA was used as an internal control to normalize ATM mRNA level. Real-time PCR quantitation of transcripts was expressed as ATM/GAPDH ratios.

### Statistical analysis

Differences between metastatic NET and non-metastatic NET samples were calculated using *t*-test or Mann-Whitney *U* test as appropriate. Correlations were examined Pearson's χ^2^, Fisher's exact test, Pearson's or Spearman's test as appropriate. Overall survival (OS) was determined using the Kaplan-Meier method and survival curves were compared using the long-rank test. Survival was calculated from the date of diagnosis and all patients were followed through March 23, 2011. All tests were two-sided and statistical significance was set at a threshold of *p*<0.05. Statistical analyses were performed using R program or SPSS (SPSS 18, Chicago, IL, USA).

## Results

### Metastatic NETs show different expression patterns of cell cycle regulatory genes

**Figure 1:**
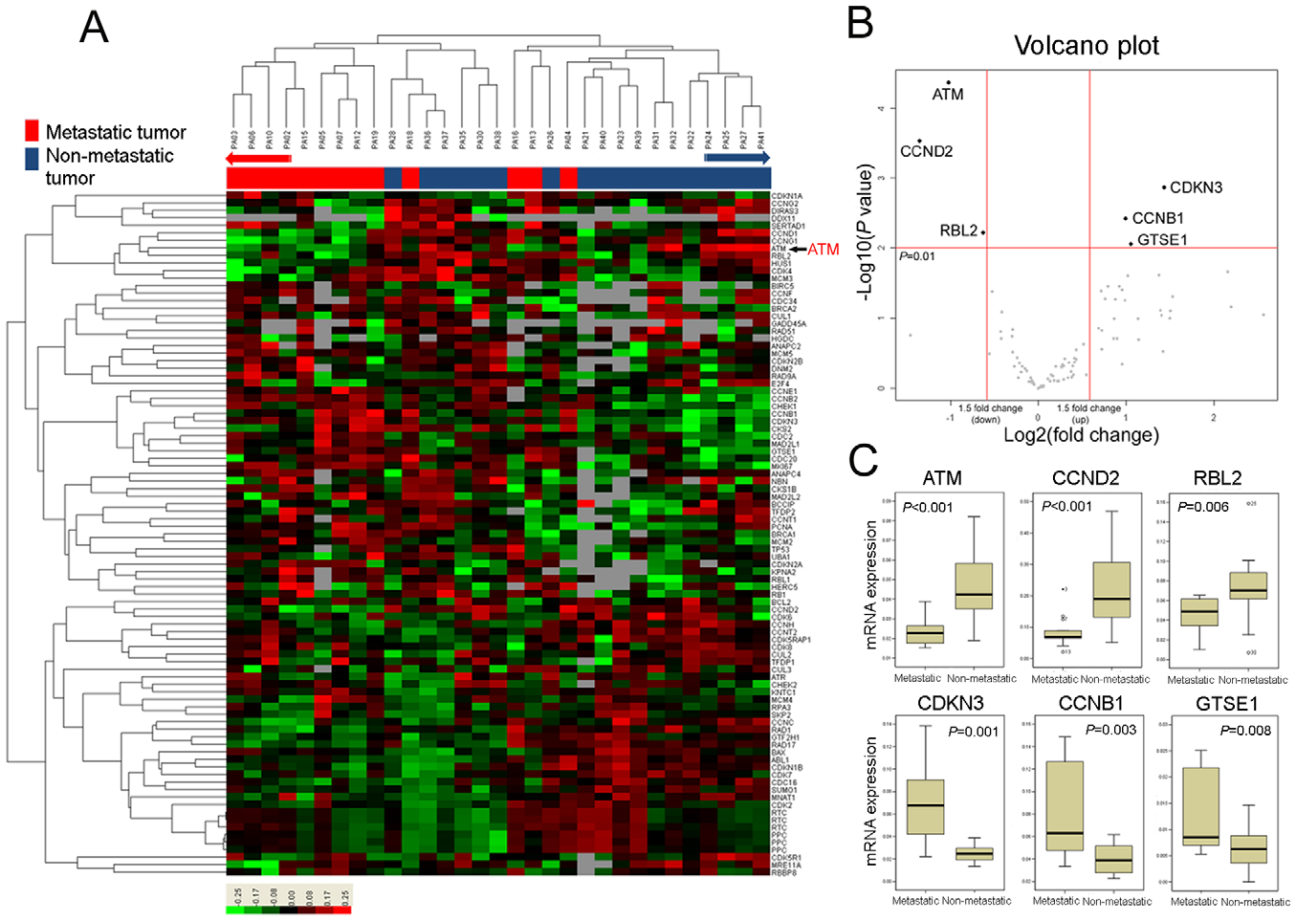
Hierarchical clustering (filtering ≥2 standard deviation) (A) and volcano plot (B) of expression data on cell cycle regulatory genes. In metastatic neuroendocrine tumors, *ATM*, *CCND2*, and *RBL2* were significantly down-regulated whereas *CDKN3*, *CCNB1*, and *GTSE1* were significantly up-regulated compared to non-metastatic neuroendocrine tumors (C).

Of 19 metastatic NETs, 13 samples passed the initial quality control for RNA and 18 of 22 non-metastatic NETs passed the quality check and thus included for cell cycle regulatory gene expression profile analysis. The experiment was repeated three times with the same result. Hierarchical clustering analysis of gene expression data (filtering ≥2 standard deviation) from all tissues revealed that metastatic NETs and non-metastatic NETs were clustered with distinctive separation at the end of both sides with few exceptions (Figure 1A). Thus, gene expression pattern of cell cycle regulatory genes in metastatic NETs was apparently distinct from that observed in non-metastatic NETs. Next step, to identify significantly different set of genes expressed between the two groups, we applied volcano plot considering both *p* value (by two-sample *t* test) and fold-changes (metastatic *vs* non-metastatic NETs). Using this method, we selected six genes including *ATM*, *CCND2*, *RBL2*, *CDKN3*, *CCNB1*, and *GTSE1* which are significantly associated with metastasis (Figure 1B). Among them, *ATM*, *CCND2*, and *RBL2* were significantly down-regulated in metastatic NETs, whereas *CDKN3*, *CCNB1*, and *GTSE1* were significantly up-regulated in metastatic NETs (all six genes, *p*<0.05, Figure 1C). Of note, ATM showed the lowest *p* value (*p*<0.001) with more than 2-fold change between non-metastatic *vs* metastatic NETs.

### Correlation analysis of six significant genes reveals that ATM plays key role as a tumor suppressor in NETs

**Figure 2:**
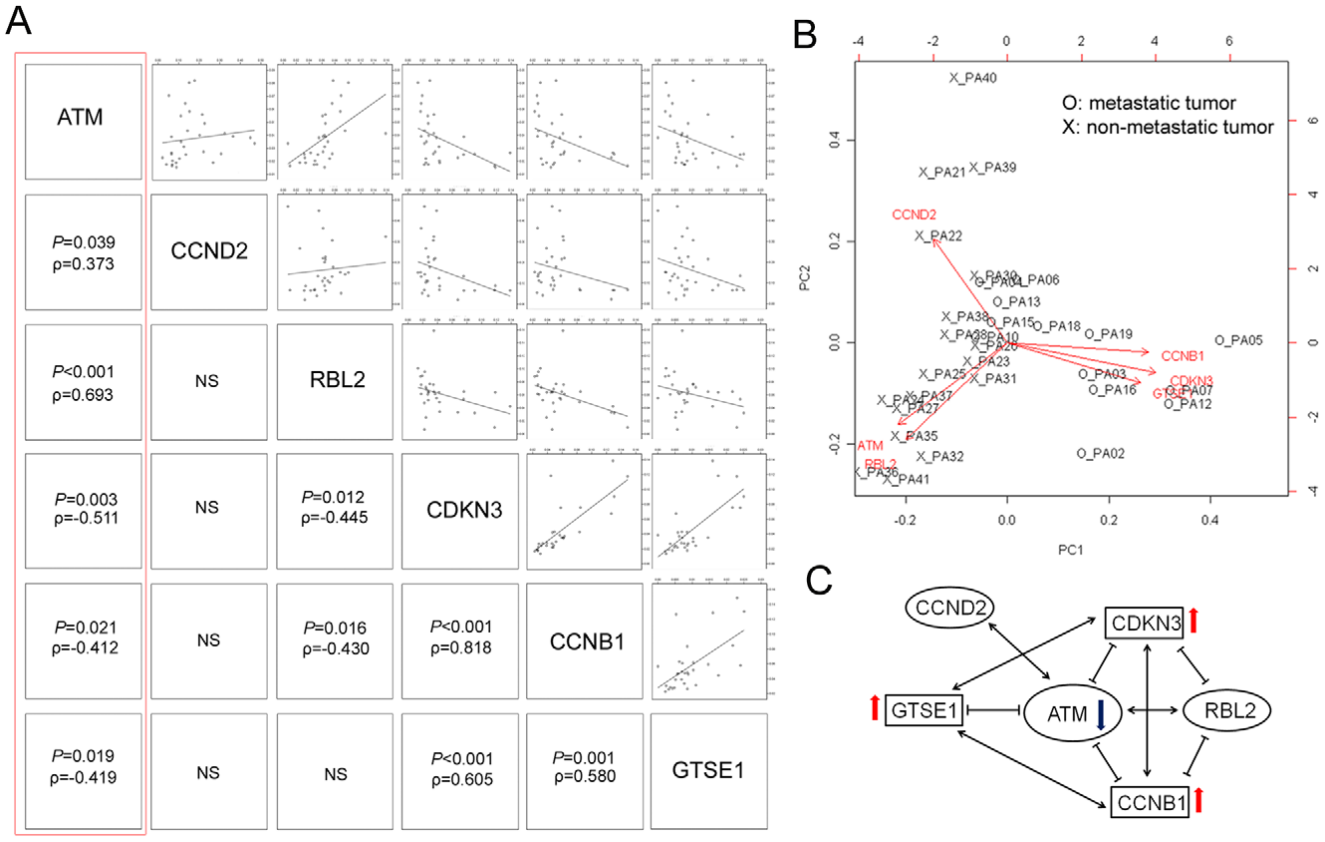
Correlation analysis of six genes reveals relationship with each other. Expression of *ATM* was only significantly associated with all other 5 genes (A). Principal component analysis showed separation of metastatic and non-metastatic neuroendocrine tumors with contributing genes (B). Network interaction diagram constructed based on correlation of six genes (C).

In the correlation analysis of six genes, only *ATM* expression was consistently correlated with expressions of all other 5 candidate genes with statistical significance (Figure 2A). *ATM* expression demonstrated strong positive correlations with *CCND2* and *RBL2*, and negative correlations with *CDKN3*, *CCNB1*, and *GTSE1*. Using these six genes, principal component analysis was performed which revealed two distinct groups of metastatic and non-metastatic NETs (Figure 2B). Furthermore, principal component analysis suggested that *CCNB1*, *CDKN3*, and *GTSE1* were important genes associated with metastatic NETs, whereas *ATM* and *RBL2* were highly expressed in non-metastatic NET with CCND2 expressed in minor group of non-metastatic NETS. *ATM* and *RBL2* are already known as negative regulators of cell cycle which support their role as tumor suppressor genes [14], [15]. In line with these findings, a network interaction diagram constructed based on correlation analyses clearly showed that an ATM down-regulation leads to increased expressions of *CDKN3*, *CCNB1*, and *GTSE1* (Figure 2C). Based on these results, ATM seems to be one of the key regulators for cell cycle in NETs.

### ATM gene down-regulation is strongly associated with metastatic NET and proliferation of tumor cells

**Figure 3:**
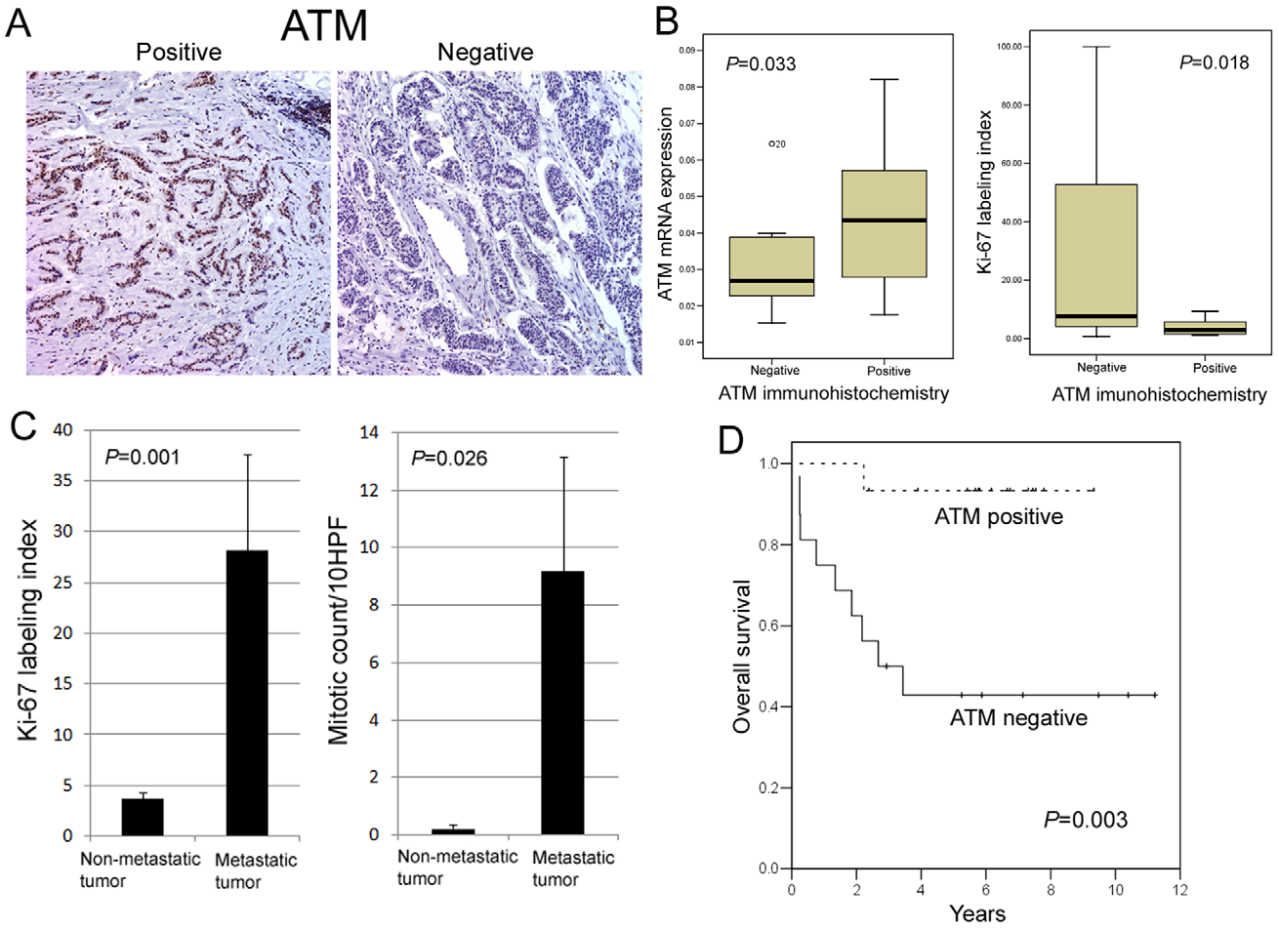
ATM immunohistochemistry in colorectal neuroendocrine tumors. ATM protein expression was significantly correlated with high ATM mRNA expression and low Ki-67 labeling index (A, B). Ki-67 labeling index and mean mitotic counts were increased in metastatic neuroendocrine tumors (C). Patients with negative ATM protein expression showed significantly shorter overall survival than patients with positive ATM protein expression (D).

To validate ATM protein expression, we performed ATM IHC from 14 of 19 metastatic NETs, and 17 of 22 non-metastatic NETs due to availability of FFPE tissue specimens. ATM IHC revealed nuclear staining of tumor cells (Figure 3A), and 15 (48.4%) of 31 cases were positive for ATM staining. The ATM positivity was defined as greater than 30% ATM (+) tumor cells in whole tumor areas. Moreover, ATM overexpression was strongly correlated with high ATM mRNA levels (Figure 3B) and low Ki-67 labeling index (Figure 3B). In line with these results, we identified that Ki-67 labeling index and mean mitotic counts were considerably increased in metastatic NETs (Figure 3C). Of 14 metastatic NETs, 12 cases (85.7%) were ATM-negative, which indicated that ATM down-regulation is strongly associated with metastatic NETs. However, there was no significant relationship between ATM expression and various clinicopathologic parameters including tumor site, grade, gender and age (Table 3). Accordingly, patients with ATM-negativity showed significantly decreased overall survival than patients with ATM-positivity (*p* = 0.003, Figure 3D), which correlated well with their metastatic behaviors.

**Table 3:** Relationships between ATM protein expression and various clinicopathologic factors in colorectal neuroendocrine tumors.

		ATM expression		
Characteristics	n	Negative	Positive	*P* value
Tumor site				
Colon	4	4	0	0.101
Rectum	27	12	15	
Grade				
G1	27	12	15	0.101
G2	4	4	0	
Sex				
Female	8	6	2	0.22
Male	23	10	13	
Age				
<60	18	9	9	0.833
≥60	13	7	6	
Tumor behavior				
Metastatic tumor	14	12	2	0.001
Non-metastatic tumor	17	4	13	

### ATM RNA expression levels in validation set

**Figure 4:**
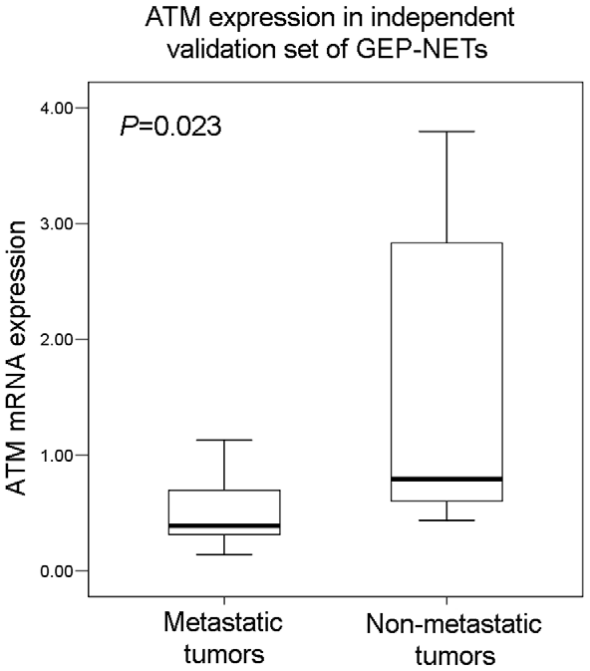
Comparison of mRNA expression levels of ATM in metastatic (mean = 0.5370±0.33263, median = 0.3912, minimum 0.14, maximum 1.13) and non-metastatic GEP-NETs (mean = 1.5075±1.22467, median = 0.7964, minimum 0.44, maximum 3.80).

In the validation set of 20 metastatic and 18 non-metastatic GEP-NETs from various organs including pancreas, stomach, ampulla of Vater and rectum, mean mRNA levels of ATM in metastatic NETs were significantly low in metastatic NETs compared to non-metastatic NETs (*p* = 0.023) (Figure 4).

## Discussion

We investigated differential gene expression signatures of metastatic *vs* non-metastatic colorectal NETs focusing on cell cycle regulatory gene set, and delineated six candidate genes for metastasis including *ATM*, *CCND2*, *RBL2*, *CDKN3*, *CCNB1*, and *GTSE1*. Among them, we have focused on *ATM* since *ATM* has been known as a tumor suppressor gene which encodes a principal DNA damage-signaling protein. In this study, decreased expression of ATM protein was strongly correlated with low ATM mRNA levels and high Ki-67 labeling index. Tumor cells with ATM dysfunction exhibit increased loss of cell cycle check points and p53 dysfunction in selected cancer types [16]–[18]. For the first time, we demonstrated here that ATM down-regulation was strongly associated with metastatic NETs when compared with non-metastatic NETs and that >80% of metastatic NETs do not express ATM protein.

In our study, low ATM mRNA expression was significantly associated with increased expression of *CDKN3*, *GTSE1*, and *CCNB1* as well as high Ki-67 proliferation index. These findings suggest that *ATM* down-regulation can lead to increased cell cycle regulatory genes such as *CDKN3* and *CCNB1*, and then increased *CDKN3* and *CCNB1* leading to increased cell division and cell proliferation. *CDKN3*, also known as kinase-associated phosphatase (KAP), is another molecule involved in cell cycle checkpoint and cell cycle arrest. It has been reported that KAP is over-expressed in breast and prostate tumors, and is associated with proliferation of tumor cells [19]. We also found that *RBL2*, a key regulator of entry into cell division was significantly down-regulated in metastatic neuroendocrine tumors, suggesting growth-suppressive properties of *RBL2* was decreased and tumor cells proloferate [20].

*ATM* gene has been described to be causal gene of Ataxia-telangiectasia, and inactivation of *ATM* is associated with increased risk of carcinogenesis and radiosensitivity [21]–[24]. Bartkova et al. [25] suggested that tumorigenic events early in the progression of major human cancer types activate the ATM-regulated checkpoint through deregulated DNA replication and DNA damage, and thereby activate an inducible barrier against tumor progression and genetic instability. Loss-of-function events in the ATM-Chk2-p53 tumor-suppressive cascade facilitates survival and proliferation of genetically unstable cancer cells [26]. Main mechanism of *ATM* inactivation in ataxia-telangiectasia associated patient is mutation such as truncating mutation and missense mutation, and missense mutation might be more prone to carcinogenesis [27]. Mutational inactivation of the *ATM* gene has been demonstrated in mantle cell lymphoma, T-cell prolymphocytic leukemias, rhabdomyosarcomas, and gastric cancer [28]–[31], and *ATM* heterozygous state is more common in breast cancer patients than in the general population [32], [33]. On the other hand, promoter methylation or deletion of *ATM* seems to be important mechanism for *ATM* inactivation in some sporadic cancers. Indra et al. [34] reported that 36% of cervical cancer samples showed *ATM* promoter methylation and 31% of cervical cancer samples showed *ATM* deletion. However, mutation in *ATM* gene was rare [35]. Ai et al. [36] also reported that frequent aberrant methylation of *ATM* promoter (25% of 100 samples) was found in head and neck squamous cell carcinomas. Both two mechanisms including methylation and deletion were well correlated with reduced mRNA and protein expression of *ATM* [34], [37]. Methylation or deletion of *ATM* may be a mechanism for reduced expression in NET, however, in our study, because of further study need to confirm.

Cell lines derived from Ataxia-telangiectasia patients exhibit abnormalities related to DNA damage and repair such as chromosomal instability, cell-cycle checkpoint defects in G1/S and G2/M, sensitivity to ionizing radiation and telomere end-to-end fusions [38]. There is a growing evidence that oral poly (ADP-ribose) polymerase inhibitor, olaparib impedes the growth of ATM null or ATM mutant lymphoma cells *in vitro* and *in vivo* by instigating the accumulation of intolerable levels of DNA damage in cycling tumor cells [39], [40]. Consistent with these reports, ATM knockdown breast cancer cells, like BRCA1/2 null cells, exhibited selective sensitivity to PARP inhibition. Given the recent positive results from phase III trial comparing conventional chemotherapy to chemotherapy plus olaparib or iniparib in breast cancer, [39], [40]. Currently, there is no standard treatment for metastatic NETs although recent trials support the use of everolimus or sunitinib as one of the treatment options [41]. In contrast to the NETs in the West, where pancreatic NET is the most prevalent NETs, colorectum is the most common anatomic tumor location in Asia [1], [42]. Hence, a clinical research with PARP inhibitor in ATM-negative colorectal NETs should be actively implemented in Asian countries.

One of the major limitations of this study was small number of tumor specimens owing to the rarity of metastatic NETs with limited availability of fresh frozen tissues for gene expression profiling. Distant metastases in rectal NETs are associated with 1.7 to 8.1% [43], and approximately 16 to 40% of colonic NETs [44]. The median survival of metastatic colorectal NETs ranges from 6 to 18 months. We showed that ATM-negativity was not significantly correlated with clinicopathologic parameters such as tumor site, histology grade, sex, and age. However, there was a tendency towards ATM negativity in colon NETs and G2 NETs when compared with rectal NETs and G1 NETS, respectively, although statistical significance was not reached (*p*>0.05).

In conclusion, our study identified six significant cell cycle regulating genes associated with metastatic colorectal NETs, and down-regulation of *ATM* and *ATM*-mediated cell cycle signaling is indicated as a key regulatory molecule leading to increased cell proliferation and involves metastasis in colorectal NETs. The anti-tumor effect of PARP inhibitor in ATM-negative, metastatic NETs should be studied in the context of clinical trials. Further studies are warranted to explain underlying biological and molecular mechanisms and develop new therapeutic targets.

## Supporting Information

Appendix S1 (DOC)
