# Supplementary material for: Metastasis of Neuroendocrine Tumors Are Characterized by Increased Cell Proliferation and Reduced Expression of the ATM Gene
Source: PLoS One. 2012 Apr 2;7(4):e34456. doi: 10.1371/journal.pone.0034456 (PMC3317775; doi:10.1371/journal.pone.0034456)
Supplement: Appendix S1 — (DOC) [file pone.0034456.s001.doc]

| **serial** | **treatment modality** | **SEX** | **AGE** | **liver mets 0= none, 1= liver mets(+)** |
| --- | --- | --- | --- | --- |
| **1** | supportive care | F | 63 | 1 |
| **2** | supportive care | F | 72 | 1 |
| **3** | supportive care | M | 71 | 1 |
| **4** | etoposide/cisplatin | M | 65 | 1 |
| **5** | supportive care | F | 64 | 1 |
| **6** | supportive care | M | 72 | 1 |
| **7** | supportive care | M | 60 | 1 |
| **8** | streptozocin/5-fluorouracil | F | 34 | 1 |
| **9** | supportive care | M | 55 | 1 |
| **10** | streptozocin/5-fluorouracil | F | 55 | 1 |
| **11** | supportive care | M | 62 | 1 |
| **12** | etoposide/cisplatin/ifosfamide | M | 63 | 1 |
| **13** | supportive care | M | 55 | 1 |
| **14** | supportive care | F | 79 | 1 |
| **15** | supportive care | M | 66 | 1 |
| **16** | etoposide/cisplatin/ifosfamide | F | 41 | 1 |
| **17** | supportive care | F | 58 | 1 |
| **18** | supportive care | M | 46 | 1 |
| **19** | supportive care | F | 59 | 1 |
